# Delirium is associated with low levels of upright activity in geriatric inpatients—results from a prospective observational study

**DOI:** 10.1007/s40520-024-02699-6

**Published:** 2024-02-14

**Authors:** Sigurd Evensen, Kristin Taraldsen, Stina Aam, Alessandro Morandi

**Affiliations:** 1 Department of Medicine, Diakonhjemmet Hospital, Oslo, Norway; 2 Department of Rehabilitation Science and Health Technology, Oslo Metropolitan University (OsloMet), Oslo, Norway; 3 Department of Geriatric Medicine, Clinic of Medicine, St. Olavs Hospital, Trondheim University Hospital, Trondheim, Norway; 4 Department of Neuromedicine and Movement Science, Faculty of Medicine and Health Service, NTNU—Norwegian University of Science and Technology, Trondheim, Norway; 5 Intermediate Care and Rehabilitation, Azienda Speciale Di Cremona Solidale, Cremona Parc Sanitari Pere Virgili, Cremona, Italy; 6 Vall d’Hebrón Institute of Research, Barcelona, Spain

**Keywords:** Delirium, Physical activity, Activity monitoring, Geriatrics

## Abstract

**Background:**

Delirium is common in geriatric inpatients and associated with poor outcomes. Hospitalization is associated with low levels of physical activity. Motor symptoms are common in delirium, but how delirium affects physical activity remains unknown.

**Aims:**

To investigate differences in physical activity between geriatric inpatients with and without delirium.

**Methods:**

We included acutely admitted patients ≥ 75 years in a prospective observational study at a medical geriatric ward at a Norwegian University Hospital. Delirium was diagnosed according to the DSM-5 criteria. Physical activity was measured by an accelerometer-based device worn on the right thigh. The main outcome was time in upright position (upright time) per 24 h (00.00 to 23.59) on the first day of hospitalization with verified delirium status. Group differences were analysed using t test.

**Results:**

We included 237 patients, mean age 86.1 years (Standard Deviation (SD) 5.1), and 73 patients (30.8%) had delirium. Mean upright time day 1 for the entire group was 92.2 min (SD 84.3), with 50.9 min (SD 50.7) in the delirium group and 110.6 min (SD 89.7) in the no-delirium group, mean difference 59.7 minutes, 95% Confidence Interval 41.6 to 77.8, *p* value < 0.001.

**Discussion:**

Low levels of physical activity in patients with delirium raise the question if immobilization may contribute to poor outcomes in delirium. Future studies should investigate if mobilization interventions could improve outcomes of delirium.

**Conclusions:**

In this sample of geriatric inpatients, the group with delirium had lower levels of physical activity than the group without delirium.

**Supplementary Information:**

The online version contains supplementary material available at 10.1007/s40520-024-02699-6.

## Introduction

Delirium is a disturbance in arousal, attention and cognition that is acute and/or fluctuating and occurs secondary to one or more physiological disturbances [1]. Delirium is common in all hospital settings [2], but is especially prevalent in geriatric patients as high age, cognitive impairment, frailty, and severe comorbidity are important risk factors [3, 4]. Delirium is distressing and consistently associated with adverse outcomes like increased mortality, increased risk of dementia, longer length of hospital stays, more complications and increased costs [5–7]. Delirium can be classified as hypoactive, hyperactive, mixed and no-subtype delirium based on highly visible psychomotor symptoms [8], and motor disturbances are suggested as core features of delirium [9].

Delirium is unrecognized in about two out of three cases [10, 11], thus, underdiagnosing may contribute to the poor outcomes of delirium [12]. Other possible contributors to the poor outcomes may be lack of cooperation with treatment and rehabilitation and that delirium initiates a negative spiral leading to complications of bedrest like pressure ulcers, hypoxia, pneumonia, and venous thromboembolism [13–15]. Hypoactive delirium is most frequently overlooked [16] and is associated with poor outcomes [17–19], the latter possibly due to complications of bedrest [13, 14].

Previous studies report that hospitalized geriatric patients on average spend between one and two hours standing or walking per day [20–23]. Patients with delirium have reduced motor function as measured by performance-based tests [9, 24, 25]. We have previously reported low levels of motor activity across all delirium motor subtypes in a sample of geriatric inpatients [26], raising the question if patients with delirium in general have low levels of physical activity contributing to underdiagnosing and poor outcomes.

The aim of this paper is to compare physical activity between patients with on-going delirium and patients without delirium in a sample of hospitalized geriatric patients using objective measures of physical activity from a body-worn accelerometer-based device. We hypothesized that patients with delirium had lower levels of physical activity than patients without delirium.

## Methods

### Design, settings and participants

This paper reports secondary analyses from a prospective observational study on delirium motor subtypes (DeMo) conducted at the medical geriatric ward at St. Olavs hospital, Trondheim University Hospital, Norway, between May 2015 and January 2017 [26–28]. St. Olavs hospital is the university hospital for the region Mid-Norway and the local hospital for the city of Trondheim and nearby municipalities.

The geriatric ward has 15 beds and is an integrated part of the Medical Clinic. Patients are admitted with a wide range of medical conditions and geriatric syndromes, except patients admitted with suspected stroke as St. Olavs hospital have a dedicated stroke unit. Patients receive comprehensive geriatric assessment and care from an interdisciplinary team of physicians, nurses, physiotherapists and occupational therapists [29]. The ward is built to enhance physical activity, and all personnel work together towards the same treatment goals including early mobilization. During the study period, there were no protocols or medical directives aiming to reduce overall physical activity of patients with delirium. The mean length of hospital stay at the ward during the study period was 7.6 days.

Acutely admitted patients ≥ 75 years were eligible for participation in the DeMo study. Exclusion criteria were inability to speak/read Norwegian and previous participation in the study. Trained nurses, occupational therapists, physiotherapists, and physicians could include patients. Participants were included within 24 h after arrival at the geriatric ward and were invited to wear an accelerometer-based device on the right thigh during hospital stay. In the present paper, we have included patients enrolled in the DeMo study with complete accelerometer data from minimum one entire day (00.00 to 23.59) with verified DSM-5 delirium status. The delirium group includes both patients with prevalent delirium on admission and patients developing delirium during hospital stay, and the non-delirium group consists of patients remaining free of delirium during the entire hospital stay.

### Ethical considerations

Due to the noninvasive, observational character of the DeMo study, patients could consent for participation and sign the consent form even if they had clear signs of cognitive impairment. For patients that obviously did not understand the information, we sought written informed consent from a proxy. When in doubt about the patient’s capacity, we always informed a proxy about the study. The project was approved by The Regional Committee for Medical and Health Research Ethics of Mid-Norway (REK Central 2015/474).

### Diagnosing delirium

Delirium was diagnosed according to the DSM-5 criteria [30]. S.E. screened all patients admitted to the geriatric ward based on chart review and interviews with proxies and staff and visited all patients with possible delirium and/or cognitive impairment. Arousal and alertness were assessed clinically and based on the first item of the Memorial Delirium Assessment Scale (MDAS), a validated scale to measure the severity of delirium [31]. Attention and cognitive function were assessed using the digit span forwards and backwards and the orientation and memory items from the MDAS. Chart review and interviews with proxies were made to ascertain that the clinical picture represented an acute change and/or fluctuation, was due to physiological disturbances and could not be explained by previous underlying cognitive impairment. All available information was considered before a decision whether the patient had delirium or not was reached. Delirium motor subtypes were classified according to the Delirium Motor Subtype Scale (DMSS) [8]. The diagnostic work-up was carried out as early as possible during the hospital stay but was not repeated daily.

### Activity monitoring

The activPAL (35 × 53 × 7 mm, 15 *g*, activPAL, PAL Technologies Ltd., Glascow, UK) is a three-axial accelerometer-based device that is worn continuously and attached with a waterproof tape to the midpoint of the thigh for the entire recording period. The activPAL utilizes the inclination of the thigh to distinguish between sitting/lying (sedentary) position and standing/walking (upright) position. The device underestimates step count in older patients due to low gait speed but is accurate in distinguishing sedentary and upright positions [32]. The activPAL focuses on positions and not intensity and is useful for research in older patients not able to perform high intensity activities [33, 34].

The ward staff member responsible for inclusion attached the activPAL device and noticed time of attachment on a paper form. Patients wore the device continuously until discharge or up to a maximum of 7 days. The device was removed before radiological procedures and reattached as soon as possible. The device was not applied in case of patient’s refusal. Regarding non-wear, all time points of removing, reattachment and final removing were recorded on the paper form. At discharge, data were downloaded through a docking station by S.E. or a trained physiotherapist who checked the quality of activity data by visual inspection of output from the activPAL software. Time in upright position per 24-h period were derived using the manufacturer’s Excel spreadsheets from software V.7.3.32 (activPAL, PAL Technologies, Ltd.) and a custom MATLAB program to create an Excel spreadsheet with activity data sorted on day 1 to day 7 for all participants.

### Outcomes

Previous studies using activPALs for activity monitoring inpatients with delirium have used sit-to-stand transitions and/or time in an upright position (upright time) as outcomes [26, 35]. In the present paper, we report upright time as minutes per day (defined as a 24-h period from 00.00 to 23.59). The main outcome is upright time the first day with complete activity data defined as no non-wear time registered on the paper form and verified delirium status, defined as day 1. The secondary outcome is upright time during the next days of the hospital stay until day 7.

### Baseline characteristics

Based on all available information, the interdisciplinary team scored the Global Deterioration Scale (GDS) as a measure of cognitive function prior to admission. If the team had not scored the GDS, S.E. completed the scale based on chart review. GDS ranges from 1 to 7, with a score of 1 indicating no signs of cognitive impairment, and a score of 7 indicating end-stage dementia [36]. We considered a score ≥ 4 as indicative of dementia. S.E. completed the Barthel Index (BI) as a measure of personal Activities of Daily Living (pADL) prior to admission based on information from proxies and electronic referrals from the municipalities. BI ranges from 0 to 20, with a score of 20 indicating independency in pADL function [37]. A now retired professor in geriatrics completed the Cumulative Illness Rating Scale (CIRS) as a measure of comorbidity [38]. CIRS ranges from 0 to 56. Zero indicates no health problems and an increasing score indicates increasing comorbidity and risk of mortality. Information on age, sex, body mass index (BMI) and place of residence were collected from the medical records.

### Statistical analyses

We present continuous data as means and standard deviations (SD) and dichotomous data as frequencies and percentages (%). Normality was checked through visual inspection of Q-Q plots. We used t test and Mann–Whitney U test to compare upright time between the delirium group and the no-delirium group on day one and linear mixed models to compare differences between the two groups across time during hospital stay. *p* values were based on two-sided tests with values < 0.05 considered statistically significant. We report 95% confidence intervals (CI) where appropriate. Power analysis was conducted for the DeMo study, but not for the analyses in the present paper. All analyses were carried out in SPSS version 27.

## Results

**Fig. 1:**
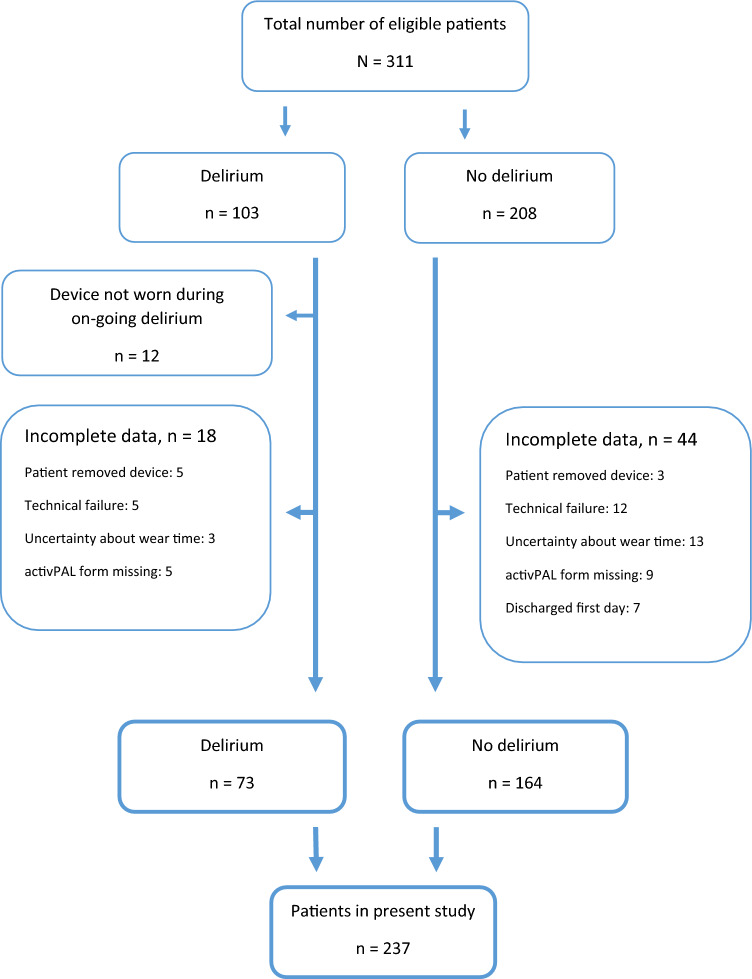
Flowchart illustrating selection of patients into the present study

In total, 311 patients were included in the DeMo study, of these 103 (33.1%) had delirium. In the present paper, we report results from 237 patients for whom we had complete activity data. Of these, 73 (30.8%) patients were diagnosed with delirium and 130 (54.8%) had a GDS-score of 4–7, indicative of dementia. Mean age was 86.1 years (SD 5.1), and 147 (62.0%) were female. Figure 1 is a flowchart illustrating the selection of patients for the present paper. Table 1 presents the participants’ baseline characteristics. Patients with delirium were more cognitively impaired, had more comorbidities and were more dependent in pADL function. Among the 73 patients with delirium, 20 (27%) had hyperactive delirium, 23 (32%) hypoactive, 20 (27%) mixed, and 10 (14%) no-subtype delirium. Baseline characteristics of the 74 patients not included in the DeMo study are described in Supplementary table S1.

**Table 1:** Baseline characteristics for all patients, the delirium group and the no-delirium group

	All (*N* = 237)	Delirium (*n* = 73)	No-delirium (*n* = 164)	Group difference *p* value^a^
	Mean (SD)			
Age (years)	86.1 (5.1)	86.8 (5.0)	85.7 (5.1)	0.15
Body mass index (kg/m^2^)	24.0 (4.2)	23.4 (3.7)	24.2 (4.4)	0.22
Cognitive function GDS^b^ score (1–7)	3.5 (1.6)	4.1 (1.4)	3.3 (1.7)	< 0.001
Comorbidity CIRS^c^ score (0–56)	13.3 (4.6)	14.3 (5.3)	12.9 (4.2)	0.046
Personal ADL^d^ function Bartel Index (0–20)	16.1 (3.6)	15.0 (4.0)	16.6 (3.3)	0.005
	Number (%)			
Female	147 (62.0)	39 (53.4)	108 (65.9)	0.07
Dementia^e^	130 (54.9)	52 (71.2)	78 (47.6)	< 0.001
Living at home	226 (95.4)	66 (90.4)	160 (97.6)	0.016

*p* values represents differences between the delirium group and the no-delirium group

^a^ *p* values were calculated by use of independent sample t test for continuous variables and Pearson’s Chi-squared test for categorical variables; ^b^*GDS* Global Deterioration Scale, ^c^ *CIRS* Cumulative Illness Rating Scale, ^d^*ADL* Activities of Daily Living, ^e^Dementia defined as GDS ≥ 4

**Fig. 2:**
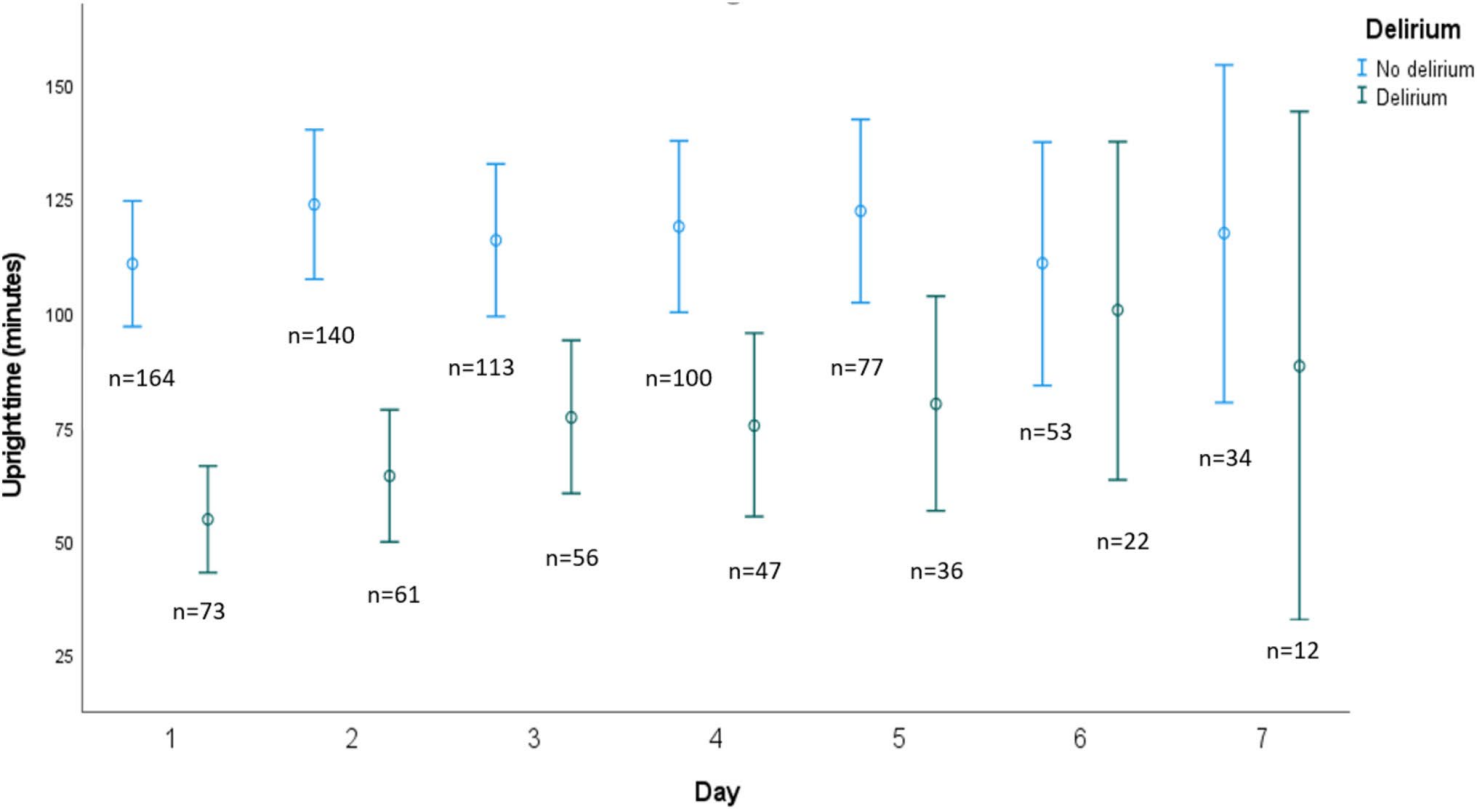
Error bars illustrating upright time in minutes as means and 95% CI for patients with and without delirium form day 1 to day 7 and number of patients in each group remaining in the study from day 1 to day 7

Mean upright time for all patients on their first day of hospital stay with complete accelerometer data was 92.2 min (SD 84.3). Patients with on-going delirium had a mean upright time of 50.9 min (SD 50.7), as compared to 110.6 min (SD 89.7) for patients without delirium, mean difference 59.7 min (CI 41.6 to 77.8). The difference was statistically significant, t (222) = 6.5, *p* value < 0.001. Repeating the comparison using Mann–Whitney U test gave similar results. In the group with delirium, upright time per day increased significantly during hospital stay, on average 6.89 min per day (*p* = 0.009), but there was no significant effect of time on upright activity in the no-delirium group (estimate 0.54 min per day, *p* = 0.77). Figure 2 illustrates upright time for the delirium group and the no-delirium group from day 1 to day 7.

Regarding delirium motor subtypes, the mean upright time for the hyperactive group was 58.3 min (SD 46.8), for the hypoactive group 40.4 min (SD 43.1), for the mixed group 35.1 min (SD 41.4) and for the no-subtype group 91.9 min (SD 46.8).

## Discussion

In this prospective observational study on acutely admitted geriatric patients, we found that patients with on-going delirium had significantly lower levels of upright activity than patients without delirium as measured objectively using an accelerometer-based device. Patients with delirium spent about half the time in an upright position as compared to patients without delirium, the difference was about one hour per day which is a large difference as the level of physical activity in hospitalized geriatric patients is generally low. The levels of upright activity between the delirium group and the no-delirium group converged towards the end of the hospital stay.

To the best of our knowledge, this is the first study comparing objective measures of motor activity between relatively large groups of acutely admitted geriatric patients with on-going delirium and patients without delirium. Godfrey compared upright activity between six patients with hyperactive delirium, ten with hypoactive, nine with mixed delirium and nine patients without delirium, reporting that patients without delirium had activity levels similar to patients with hyperactive delirium [35]. Bellelli investigated the relation between delirium and physical performance in an in-hospital rehabilitation unit and reported that patients with on-going delirium had poorer results on physical performance tests as measured with the Tinetti scale and the Trunk Control Test than patients without delirium, and that results improved when delirium resolved [24]. Gual found that patients with delirium on admission to acute and rehabilitation wards had reduced motor functions on the physical performance test Hierarchical Assessment of Balance and Mobility (HABAM), this was particularly pronounced for patients with delirium superimposed on dementia [25]. Richardson reported that among acutely hospitalized older patients assessed daily for delirium and physical function with the HABAM scale, patients with delirium had poorer physical function than patients without delirium [9]. Put together, these three studies strongly indicate that patients with delirium have reduced motor function as measured by physical performance tests [9, 24, 25]. Our results add that reduced motor function translates into reduced levels of upright activity, supporting the argument that motor disturbances are core features of delirium.

According to our results, delirium means less, not more, physical activity, which is an important message to all clinicians and delirium researchers, both in terms of delirium recognition and delirium care. Delirium may remain underdiagnosed because patients presenting with hypoactive symptoms are not recognized with delirium [13, 14], and incorporation of motor disturbances in delirium screening tools may contribute to increased delirium recognition since about 90 % of patients with delirium have some sort of motor symptoms [14, 17–19, 27]. The results also suggest that acutely impaired gait function and tendency of falling could be signs of delirium and among other interventions should call for delirium screening.

Several studies document low levels of ambulation and physical activity among hospitalized older patients [20, 21, 23, 39], and low levels of in-hospital physical activity is associated with elevated mortality [22]. Delirium affects one in four hospitalized older patients [40], and our results indicate that all patients with delirium, not limited to the hypoactive group, have very low levels of physical activity. Low levels of physical activity may contribute to a decline in muscular strength, balance and gait function and reduce out of bed activities like dressing, hygiene and eating, all key activities to live independently. At worst, low levels of physical activity may lead to life threatening complications of bedrest like infections, hypoxia and venous thromboembolism, and it is possible that bedrest linked to delirium contributes to poor outcomes like increased length of hospital stay, institutionalization and increased mortality. Consequently, physical activity is endorsed in the delirium management guidelines and should be provided by a multidisciplinary team. Specifically, Inouye and colleagues have created a mobility action package to promote mobilization and walking during an acute hospital stay, and current knowledge highlight the importance of incorporating such mobility programs in delirium care [41].

In line with previous studies [9, 24, 25], patients diagnosed with delirium increased their activity levels towards end of hospitalization. This is likely due to delirium resolution but remains uncertain as we did not conduct repeated delirium assessment. The no-delirium group does not seem to show the same increase in upright time, this could be due to a selection bias as the fittest patients without delirium likely were discharged early and the patients remaining in-hospital towards day 7 show slow recovery from their illness.

Our results and previous studies on motor activity and delirium call for studies investigating how motor disturbances could be incorporated into delirium screening programs and for well-conducted mobilization trials investigating if interventions focusing on physical activity could improve outcomes for patients with delirium. Further, there is no established method to monitor delirium over time and no consensus on how to decide when a delirium episode has resolved. Repeated measures of physical function may be a strategy for delirium monitoring [9, 24, 25], our results raise the question if “live” activity monitoring with small devices could be an alternative for activity monitoring in general and delirium monitoring specifically [42]. Compared to repeated assessment by health care personnel such monitoring may be time-sparing and more feasible. Finally, recent studies highlight the importance of integrating artificial intelligence and machine learning algorithms in geriatric medicine [43]. Indeed, automatic monitoring of motor changes during hospitalization may support early recognition of delirium and its evolution improving rapid and accurate treatment of the underlying causes.

The strengths of this study are the objective measures of physical activity and the relatively large sample size. Limitations are the lack of repeated delirium assessment, the lack of measures of physical function prior to admission and the lack of frailty assessment. The differences in activity levels could be explained by differences in baseline characteristics, but we have previously published results indicating that cognitive function, age, comorbidities and dependency do not influence physical activity in hospitalized geriatric patients [23]. Further limitations are the lack of admittance diagnosis and uncertainty about the quality of non-wear registrations that could have influenced the activity data from day 2 to day 7. However, a very low percentage of the patients has registered non-wear in this period, ranging from 6.3% on day 2 to 0 on day 7 with limited impact on the results. A possible limitation is that we report secondary analysis from a study designed for other purposes, but the DeMo study was a dedicated delirium study, and activity monitoring during hospital stay was an integrated, pre-planned part of the study design.

To conclude, hospitalized geriatric patients with on-going delirium had significantly reduced upright activity as compared to patients without delirium; the latter group spent more than twice the time daily in upright position as compared to the delirium group. The low levels of upright activity in the delirium group are of particular concern since hospitalization by itself carries a large risk of immobilization and loss of physical function. Findings support that motor disturbances are core features of delirium that could be used to improve the detection of delirium and raise the question whether low levels of upright activity may contribute to poor outcomes of delirium. The results emphasize the need for further research investigating if incorporation of motor disturbances in screening tools could improve delirium detection and if mobilization interventions could improve delirium outcomes.

### Supplementary Information

Below is the link to the electronic supplementary material.

Supplementary file1 (DOCX 13 KB)

## Data Availability

Data are not available as we do not have approval from The Regional Committee for Medical and Health Research Ethics of Mid-Norway to share data.
