# Supplementary material for: Delirium is associated with low levels of upright activity in geriatric inpatients—results from a prospective observational study
Source: Aging Clin Exp Res. 2024 Feb 14;36(1):41. doi: 10.1007/s40520-024-02699-6 (PMC10867047; doi:10.1007/s40520-024-02699-6)
Supplement: Supplementary file 1 — Supplementary file1 (DOCX 13 KB) [file 40520_2024_2699_MOESM1_ESM.docx]

**SUPPLEMENTARY MATERIAL**

**Supplementary table S1**. Baseline characteristics for the 74 patients included in the DeMo study, but excluded from the present study.

|  | **Mean (SD)** |
| --- | --- |
| **Age** (years) | 86.4 (6.1) |
| **Body mass index** (kg/m^2^) | 24.6 (4.7) |
| **Cognitive function** GDS^a^ score (1-7) | 3.5 (1.8) |
| **Comorbidity** CIRS^b^ score (0-56) | 13.0 (4.9) |
| **Personal ADL^c^ function** Bartel Index (0-20) | 15.9 (4.6) |
|  |  |
|  | **Number (%)** |
| **Female** | 38 (51.4) |
| **Dementia^d^** | 39 (52.7) |
| **Living at home** | 68 (91.9) |

Notes ^a^GDS: Global Deterioration Scale; ^b^CIRS: Cumulative Illness Rating Scale; ^c^ADL: Activities of Daily Living; ^d^Dementia defined as GDS ≥ 4.
